# Immunogenicity of MultiTEP platform technology-based Tau vaccine in non-human primates

**DOI:** 10.1038/s41541-022-00544-3

**Published:** 2022-10-12

**Authors:** Armine Hovakimyan, Karen Zagorski, Gor Chailyan, Tatevik Antonyan, Levon Melikyan, Irina Petrushina, Dash G. Batt, Olga King, Manush Ghazaryan, Aashrit Donthi, Caitlynn Foose, Nikolai Petrovsky, David H. Cribbs, Michael G. Agadjanyan, Anahit Ghochikyan

**Affiliations:** 1 Department of Molecular Immunology, Institute for Molecular Medicine, Huntington Beach, CA USA; 2 Institute for Memory Impairments and Neurological Disorders, University of California, Irvine, Irvine, CA USA; 3 Charles C. Gates manufacturing Facility, University of Colorado/Anschutz Medical Campus, Aurora, CO USA; 4 Vaxine Pty Ltd, Flinders Medical Center, Bedford Park, Adelaide, SA 5042 Australia; 5 Department of Diabetes and Endocrinology, Faculty of Medicine, Flinders University, Adelaide, SA 5042 Australia

**Keywords:** Protein vaccines, Protein vaccines

## Abstract

Pathological forms of Tau protein are directly associated with neurodegeneration and correlate with Alzheimer’s Disease (AD) symptoms, progression, and severity. Previously, using various mouse models of Tauopathies and AD, we have demonstrated the immunogenicity and efficacy of the MultiTEP-based adjuvanted vaccine targeting the phosphatase activating domain (PAD) of Tau, AV-1980R/A. Here, we analyzed its immunogenicity in non-human primates (NHP), the closest phylogenic relatives to humans with a similar immune system, to initiate the transition of this vaccine into clinical trials. We have demonstrated that AV-1980R/A is highly immunogenic in these NHPs, activating a broad but unique to each monkey repertoire of MultiTEP-specific T helper (Th) cells that, in turn, activate B cells specific to PAD. The resulting anti-PAD IgG antibodies recognize pathological Tau tangles and Tau-positive neuritis in AD case brain sections with no staining in control non-AD cases. These published data and efficacy results support the AV-1980R/A vaccine progression to first-in-human clinical trials.

## Introduction

Alzheimer’s disease (AD) is the most common cause of dementia and may contribute to 60-70% of dementia cases. According to the WHO, an estimated 55 million people live with dementia worldwide, and there are 10 million new cases yearly^1^. The enormous and increasing worldwide healthcare burden due to AD (by 2050, the cost of treatment in the US is expected to rise to $1.1 trillion a year) combined with a lack of effective interventions indicate that new disease-modifying approaches for AD treatment are essential. AD is a complex and multifactorial disease, and the mechanisms underlying the pathophysiology of this disease are still unclear. Nevertheless, over the last three decades, the “amyloid cascade hypothesis” has been proposed and amended as a potentially unifying theory of AD, with the premise that Aβ accumulation represents the initiating toxic event triggering a neurodegenerative cascade that involves amyloid deposition, followed by Tau hyperphosphorylation, inflammation, oxidative stress, synaptic and neuronal loss^2–5^. Not surprisingly, the development of potential therapies for AD has been focused mainly on reducing pathological Aβ or Tau and, more recently, on the inflammation associated with the accumulation of these pathological molecules in the brain.

Immunotherapy has been considered one of the most promising strategies to reduce abnormal Aβ or Tau levels in the brain. However, despite early optimism from active and passive immunization trials targeting Aβ and Tau, these approaches have thus far failed to slow disease progression in AD patients and even in MCI subjects significantly^6–15^, likely because the treatment was initiated too late^16,17^. For example, a high dose of monoclonal antibody (mAb), aducanumab targeting the N-terminal B cell epitope of Aβ, significantly reduced the Aβ plaques but did not show significant clinical benefit in early AD subjects. Nevertheless, it received the FDA accelerated approval based on Aβ plaque reduction^18^. Similarly, an extremely high dose of gosuranemab (Bristol-Myers Squibb/iPierian) specific to the N-terminal region of extracellular Tau (eTau) decreased over 90% eTau in the CSF in subjects with progressive supranuclear palsy^19^ and mild cognitive impairment due to AD or mild AD but unfortunately, showed no efficacy in both PASSPORT and TANGO trials^20^. Recently, AC Immune announced that semorinemab, targeting eTau, significantly reduced the rate of decline in ADAS-Cog11 by 43.6% compared with the placebo in mild-to-moderate AD patients^21^. Although these results are promising, trials showed no benefit for the other cognitive or functional outcomes. Therefore, current scientific discussions on Aβ and Tau immunotherapy are centered on the following major topics: (i) preventive immunization (once/twice per year) with an immunogenic active vaccine vs. frequent (weekly/monthly) administrations of extremely high doses of mAb and (ii) selection of disease-related B cell epitopes for active vaccination; (iii) generation vaccine targeting post-translationally modified (pyroglutamate-modified Aβ and Tau phosphorylated at specific residues).

Our long-standing tenet based on preclinical data^22,23^ suggests that immunizations of cognitively unimpaired people at risk for disease by a safe and immunogenic human vaccine could delay the onset of AD. Accordingly, we have developed a universal vaccine platform (MultiTEP) for neurodegenerative disorders, which is designed to (i) overcome self-tolerance by inducing Th cell responses to MultiTEP, but not to self-epitopes of endogenous molecules (e.g., Aβ/Tau/α-Synuclein); (ii) diminish variability of immune responses stemming from the HLA diversity in humans; (iii) augment the antibody production through activation of not only naïve Th cells but also pre-existing memory Th cells, which will be especially beneficial for elderly patients with immunosenescence^24,25^. Our MultiTEP platform-based AV-1980R vaccine (Supplementary Fig. 1) targeting the N-terminal region of Tau (spanning aa 2-18, Tau_2-18_) induced high titer of anti-Tau antibodies in wild-type, Tau/Tg (PS19 and rTg4510), and Thy-Tau22/5XFAD (T5x) bigenic mice without the activation of autoreactive Tau-specific T cells. Vaccine-induced anti-Tau antibodies reduced the total Tau and various phosphorylated Tau species in the brains of immunized mice and improved their cognitive and motor functions^26–29^. Importantly, purified antibodies specific to Tau_2-18_ did not recognize native Tau^27,30^.

To support the transition of this preventive vaccine toward human clinical trials, here we report on the cGMP (engineering run) manufacturing of recombinant vaccine. We then provide data on the immunogenicity and overall safety of the AV-1980R/A vaccine evaluated in non-human primates (NHP), the closest phylogenic relatives to humans with a comparable immune system.

## Results

### Production and characterization of purified cGMP grade recombinant protein, AV-1980R

The AV-1980R recombinant protein was purified from inclusion bodies expressed in *E. coli* BLR(DE3) cells (see Materials and Methods). We solubilized inclusion bodies in 8 M urea and purified them under denaturing conditions. To prevent the formation of disulfide bonds, we used sulfone-based protection of thiols followed by reducing agent deprotection after initial purification. Further purification steps include membrane filtration, precipitation using an ammonium sulfate solution, ion-exchange chromatography with elution from a Q-Sepharose followed by SP-Sepharose resins, and ultrafiltration. Finally, the protein was transferred to a non-denaturing environment by diafiltration using buffer exchange at a controlled rate to generate particles of reproducible size and population distribution (see details in Methods).

**Fig. 1:**
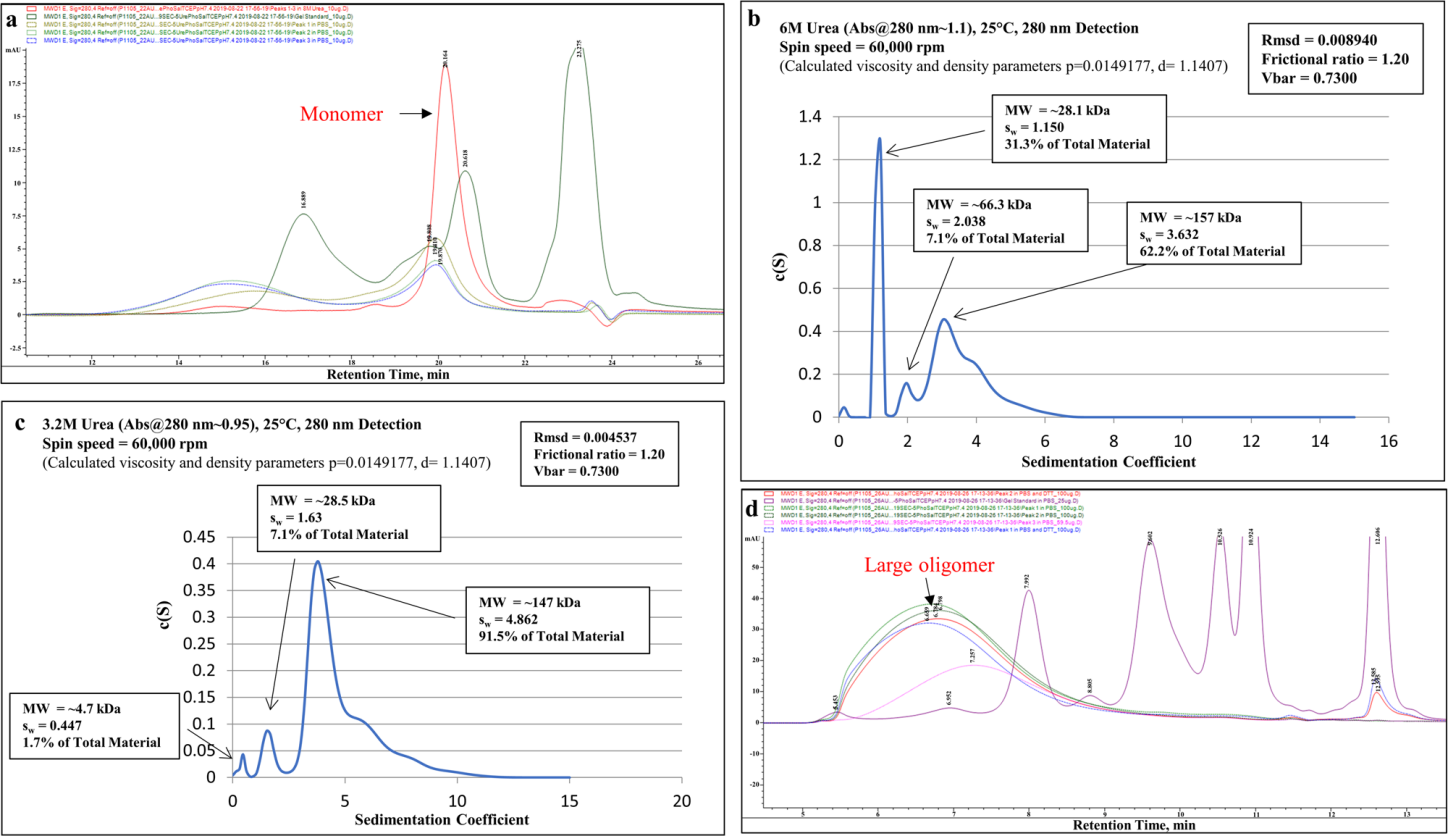
Characterization of the bulk AV-1980R product during manufacturing. **a** size exclusion chromatography (SEC) analysis of the AV-1980R protein in 8 M urea in the mobile phase. **b** Analytical ultra-centrifugation (AUC) profiles of the protein in 6 M urea. **c** AUC profiles of the protein in 3.2 M urea. **d** SEC analysis of the protein with no urea in the mobile phase.

**Fig. 2:**
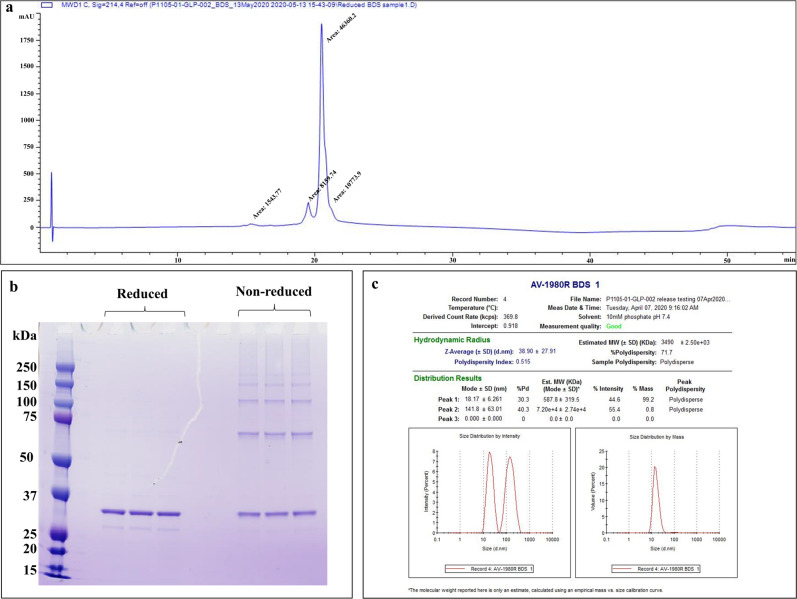
Characterization of purified AV-1980R protein by RP-UPLC, SDS-PAGE assay, and DLS profile. **a** Representative chromatogram from reverse-phase ultra-performance liquid chromatography (RP-UPLC): C4 2.1 x 50 mm, 1.7 μm column; Mobile Phase A: 0.1% TFA in water; Mobile Phase B: 0.1% TFA in acetonitrile; Flow rate: 0.5 mL/min, Gradient 0–100%B over 37 min. **b** SDS-PAGE profiles of the AV-1980R protein in reduced and non-reduced conditions. The main band in all samples corresponds to the expected molecular weight of AV-1980R of 31.6 kDa. The numbers on the left side of the image correspond to the molecular weight of the standard in kilodaltons. **c** DLS profile of AV-1980R, distribution by mass and intensity.

In size exclusion chromatography (SEC) with an 8 M urea mobile phase, the purified protein was highly monomeric/monodisperse (Fig. 1a). The gradual decrease in urea concentration led to protein aggregation, and most of the protein population was in a monomeric-dimeric-tetrameric state in 6 M urea (Fig. 1b) and mainly in a tetrameric state in 3.2 M urea (Fig. 1c). In the mobile phase without urea, protein self-association proceeded further, and it appeared in oligomeric forms with molecular weights >670 kDa (Fig. 1d). The reduced RP-UPLC analysis of AV-1980R shows that it elutes mainly as a single, monodisperse peak (Mean % main peak: 69.4%, retention time: 20.4 min) with a small pre-main peak (12.2%, lower aggregated form retention time: 19.5 min) and a post-peak shoulder (16.1%, higher aggregated form retention time: 21–21.5 min) (Fig. 2a). SDS-PAGE analyses of the protein in reduced conditions revealed the major band identified at ~32 kDa, matching the theoretical molecular weight of 31.6 kDa, and minor bands of higher molecular weight corresponding to oligomers of different sizes. In non-reducing conditions, a shift from the monomer to higher molecular weight oligomers occurred (Fig. 2b). Dynamic Light Scattering (DLS) analysis showed the polydisperse nature of the oligomerized protein (PDI = 0.515). However, intensity and mass distribution results showed that 99.2% of particles are, on average, 18.17 ± 6.261 nm in diameter with a molecular weight of 587.8 ± 319.5 kDa, suggesting a uniform size distribution (Fig. 2c). The highly aggregated population (141.8 ± 63.01 nm, MW 7.2e + 4 ± 2.74e + 4) makes up only 0.8% of the protein, and the signal is not visible in mass distribution. The protein was stable after several freeze/thaw cycles, with similar DLS results. The presence of residual endotoxin (254 EU/mg), host cell DNA (117 ng/mg), and host cell protein (111.5 ng/mg) in the final material is shown to be in the range required by the FDA. We demonstrated that purified AV-1980R protein is stable at −20 °C for up to 12 months and −80 °C for up to 18 months (Table S1), and we continue to check the stability periodically. After thawing, the protein is also stable at 25 °C for up to 72 h and 2–8 °C for up to 1 month (Table S1). Considering that the protein should be mixed with Advax^CpG^ adjuvant^31–33^ at the bedside before administration to people, we also checked the stability of AV-1980R formulated in Advax^CpG^ and showed that this vaccine (AV-1980R/A) is stable at room temperature for up to 72 h (Supplementary Fig. 2).

### Immunogenicity in non-human primates

To evaluate the immunogenicity and the overall safety of the putative human vaccine AV-1980R/A candidate, we decided to use NHP with an immune system similar to humans. More specifically, although the sequences and effector functions of humans and NHP antibodies are not the same^34^, they both possess highly polymorphic MHC class II molecules^35^. Accordingly, we evaluated the immunogenicity of the AV-1980R/A vaccine in 12–18 years old NHPs (*Macaca fascicularis)*, showing 98% Tau homology with humans. The scheme of the immunizations is shown in Supplementary Fig. 3.

**Fig. 3:**
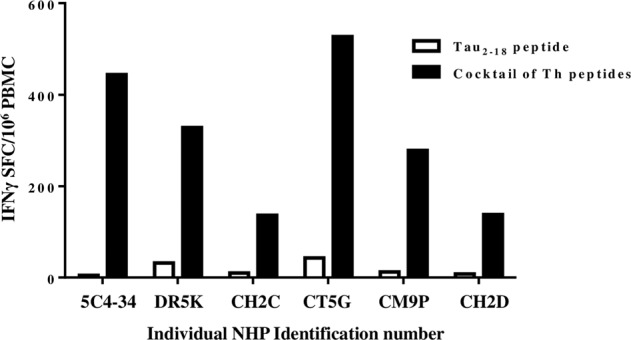
AV-1980R/A induced strong cellular responses specific to MultiTEP Th epitopes but not Tau_2-18_ peptide in adult non-human primates. INFγ producing T cells were detected in PBMC of vaccinated NHPs by ELISPOT assay. The individual monkeys are plotted on the x-axis, and the number of INFγ-positive T cells is plotted on the y-axis. The PBMC restimulation is done by either the cocktail of peptides derived from the MultiTEP platform (black bars) or by the Tau_2-18_ peptide (white bars).

First, we measured the cellular immune responses after re-stimulating PBMC with a cocktail of promiscuous Th cell peptides incorporated into the MultiTEP platform or with the Tau_2-18_ peptide. Of note, the sequence of this region in NHP Tau differs from human Tau only by one residue, the aspartic acid in NHP and glutamic acid in humans. As expected from our previous monkey studies with MultiTEP-based vaccine targeting Aβ^36^, the immunizations with AV-1980R/A anti-Tau vaccine induced a strong cellular immune response specific to MultiTEP but not to Tau_2-18_ in every monkey (Fig. 3). Vaccination generated the highest cellular immune responses in 2 monkeys (animal ID 5C4-34 and CT5G): over 400 IFNγ SFC per 10^6^ were detected. In the other four animals, the number of MultiTEP-specific Th cells in PBMC was lower: over 220 IFNγ SFC per 10^6^ PBMC in CHD2 and CH2C and over 100 in DR5K and CM9P.

**Fig. 4:**
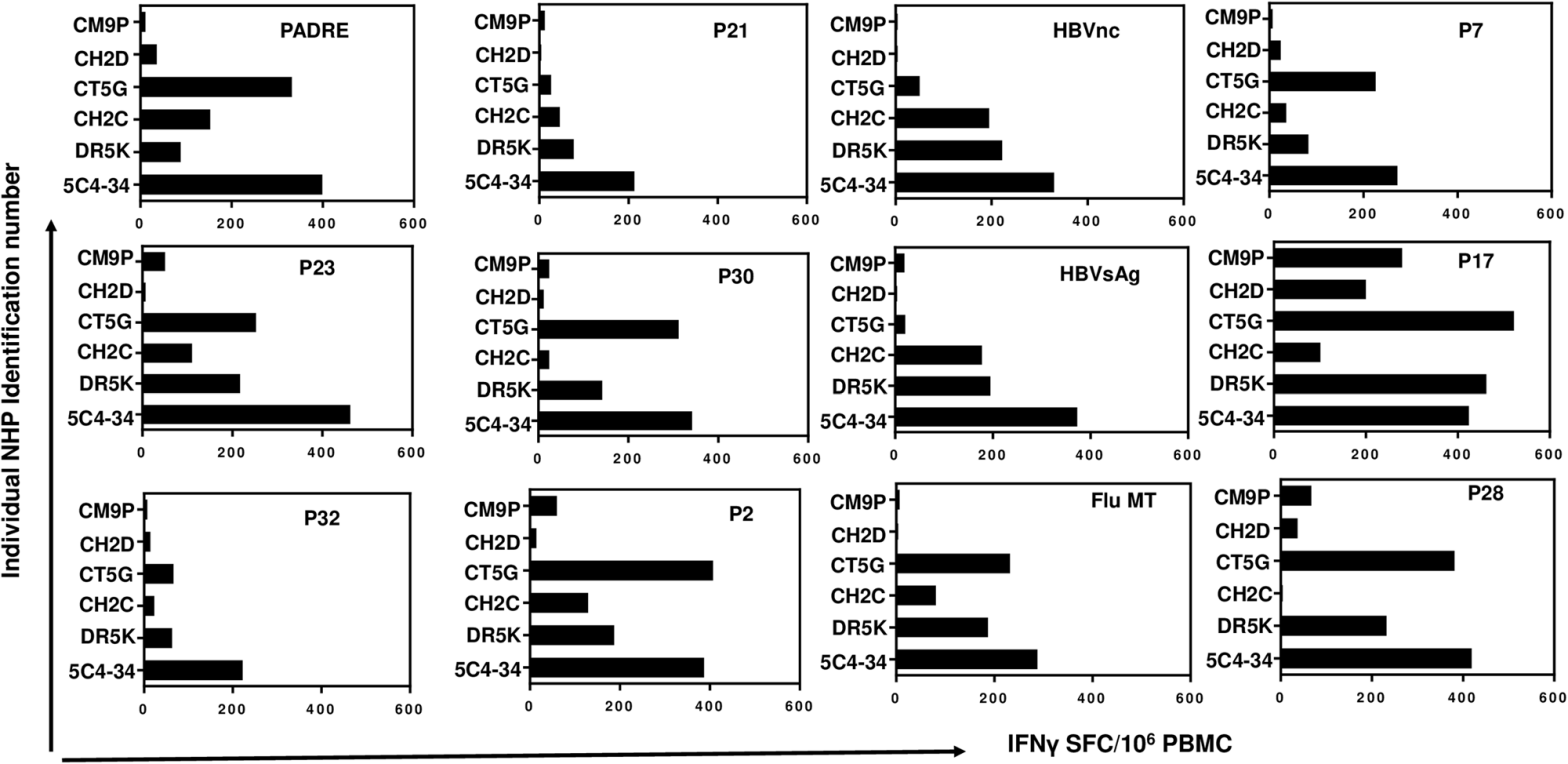
AV-1980R/A activated a broad repertoire of Th cells specific to individual Th epitopes comprising MultiTEP in NHPs. Data presented as the number of IFNγ cytokine-producing cells (SFCs) specific to individual peptides minus the background level. All peptides were used at 20 μg/ml.

To determine the epitopes recognized by the MultiTEP-specific Th cells in the immunized macaques, we stimulated PBMC of individual animals with each peptide incorporated in the vaccine platform separately (Fig. 4). The quantitative analyses demonstrated that while the AV-1980R/A vaccine activates an extensive repertoire of Th cells specific to MultiTEP in all monkeys, each animal responds to a particular set of epitopes. In other words, the same Th epitope within the MultiTEP could stimulate strong, mediocre, weak, and even no cellular responses in different monkeys (Fig. 4, Supplementary Table 2). For example, we detected strong cellular responses specific to P28 (~400 IFNγ-positive SFCs per 10^6^ PBMCs) in two animals. The response was mediocre (~230 IFNγ-positive SFCs per 10^6^ PBMCs) in one macaque, weak (30–60 IFNγ-positive SFC on 10^6^ PBMCs) in 2, and one animal did not respond to it at all (Fig. 4, Supplementary Table 2). Taken together, analysis of Th cell epitopes in vaccinated monkeys demonstrated the activation of a broad, individualized repertoire of MultiTEP-specific Th cells. Based on these data, we anticipate that AV-1980R/A can stimulate immune responses in most, if not all, vaccinated people regardless of the MHC class II gene polymorphism.

**Fig. 5:**
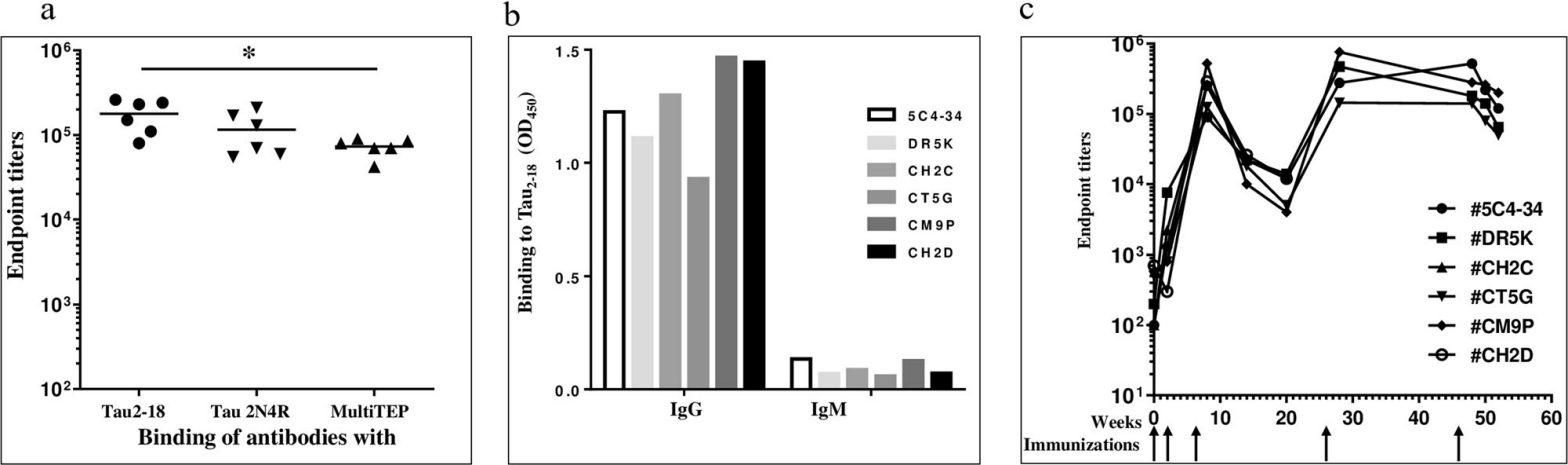
AV-1980R/A induced high titers of anti-Tau antibodies in Macaca fascicularis. **a** Endpoint titers of the generated antibodies were evaluated in the sera of individual animals after three immunizations. Horizontal lines indicate the median titer and the x-axis grouping indicates the target-specificities of the generated responses. Titers of generated antibodies specific to MultiTEP are significantly lower than titers of antibodies specific to Tau2-18 (*p = 0.0173, Ordinary One-way Anova) (**b**) AV-1980R/A induced IgG isotypes of antibodies. Isotypes were analyzed in sera of individual animals diluted 1:2000 after the third immunization. **c** Dynamics of humoral immune responses in individual animals (*n* = 6 at the start, #CH2C was euthanized on week 12, and #CH2D was euthanized on week 24) immunized with AV-1980R/A on weeks 0, 2, 6, 26, and 46.

These overall strong MultiTEP-specific cellular immune responses (Fig. 3 & 4, Supplementary Table 2) supported the induction of high titers of anti-Tau antibodies in all vaccinated NHPs (Fig. 5a). An average concentration of antibodies calculated based on anti-Tau2-18 humanized mAb was 232±67µg/ml. Next, the IgG and IgM isotypes of Tau-specific antibodies in pre-bleed and immune serum samples of NHP were evaluated. Data demonstrated that all the vaccinated animals produced IgG antibodies specific to human Tau protein, with negligible IgM (Fig. 5b), indicating that the humoral immune responses were Th cell-dependent. The dynamics of IgG antibody responses showed that they peaked after the third immunization, then slightly decreased throughout the twenty weeks. The fourth immunization at week 26 boosted the antibody production, which persisted at the same level up to week 55 (Fig. 5c). Each animal was observed daily for abnormal appearance and behavior by the veterinarian. In addition, the cage floor and other structures were scanned for blood, evidence of birth, diarrhea, etc. All animals were visually monitored for signs of diarrhea, dehydration, cuts, lacerations, etc. There were no differences in general health status between experimental and control groups during five vaccine administrations and a one-year follow-up.

### Specificity of anti-Tau antibodies

**Fig. 6:**
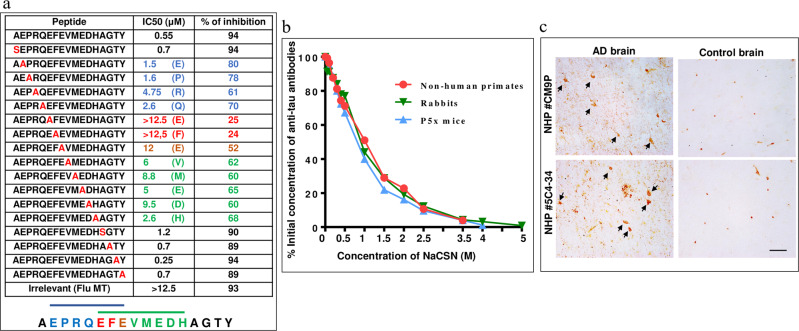
Characterization of antibodies induced by vaccination with AV-1980R/A in NHP. **a** Epitope mapping of immune sera from vaccinated Macaca fascicularis was performed by alanine scanning competition ELISA. Two overlapped epitopes have been detected comprising amino acids 3–9 EPRQEFE and amino acids 7–14 EFEVMEDH. IC_50_ and the percent of inhibition of antibody binding to Tau_2–18_ peptide with mutated peptides (alanine substitution of each single amino acid) is shown in the table. **b** Relative avidity for binding with Tau protein in mice, rabbits, and monkeys was determined by Sodium thiocyanate displacement ELISA using purified antibodies from pooled sera of individual animals. The effective concentration of NaSCN required to release 50% of primary antibodies from the ELISA plate (half-maximal effective dose (ED_50_) was 0.8 M for mice, 0.9 M for rabbits, and 1 M for NHP. The concentration of antibodies was adjusted to 30 ng/ml. **c** Immune sera, but not pre-bleed sera bound to neurofibrillary tangles (NFT) in brain sections from the AD case. Sera were diluted at 1:1000. The original magnification is 40X, and the scale bar is 20 μm. Arrows show NFTs.

To define the specificity of antibodies induced in NHP, we performed the fine mapping of the B cell epitopes of the antibodies purified from the immune sera of AV-1980R/A vaccinated macaques. The vaccine-induced anti-PAD antibodies were blocked by 94% with Tau-2-18 peptide, while peptides mutated at positions 4–14 amino acids decreased the blocking of antibodies to different degrees (Fig. 6a). Thus, mapping showed that generated antibodies were specific to two epitopes of Tau that share three critical amino acid residues: _7_EFE_9_. These three residues of Tau_2-18_ were essential for the antibodies generated in mice vaccinated with AV-1980R/A^26^, and we next compared the relative avidity of IgG purified from the pooled sera of vaccinated NHP, T5x bigenic mice, and rabbits immunized with this vaccine. The concentration of sodium thiocyanate required to dissociate 50% of the antibodies (half-maximal effective dose, ED50) was 1 M for NHPs, 0.9 M for rabbits, and 0.8 M for mice, indicating that the avidity of antibodies generated in NHP was slightly higher than that of the antibodies generated in rabbits and mice (Fig. 6b).

Finally, we showed that NHP antibodies generated after vaccinations with AV-1980R/A bind neurofibrillary tangles and neuropil threads in AD case brain sections but not in control non-AD brain sections (Fig. 6c).

## Discussion

AD is a complex disease involving various genetic and environmental risk factors that lead to the development of two hallmark pathologies: Aβ oligomers/fibrils/plaques and Tau aggregates/tangles, followed by inflammation and severe neurodegeneration. The Aβ deposition occurs independently in the neocortex and induces or facilitates the spread of pathological Tau, perhaps by promoting the production of pathological Tau forms^37^. According to the modified amyloid cascade model^3–5^, primary age-related amyloidosis develops universally as a function of aging but produces no or only mild cognitive symptoms. Thus, in this AD model, Aβ does not directly cause cognitive symptoms but is central to the disease pathogenesis as a dominant driver of downstream pathological processes^3–5^. Importantly, recent longitudinal analyses of participants within the AD Neuroimaging Initiative (ADNI) demonstrated evidence for synergism between Aβ and Tau, suggesting that these pathologies may interact to trigger the progression from amnestic mild cognitive impairment (MCI) to AD dementia^3–5,38–40^.

As mentioned above, clinical trials with active and passive immunotherapies targeting Aβ and Tau neuropathology have not been successful despite reducing the pathological molecules. For example, the fully human anti-Aβ mAb aducanumab, which recently obtained accelerated approval from the FDA, significantly reduced Aβ accumulation in subjects but did not improve cognition or prevent the disease manifestation^18,41,42^. Tau immunotherapies have also been proposed based on the preclinical studies^43–45^, and they quickly moved into clinical trials^45–48^ as disease-modifying treatments of dementia associated with various Tauopathies, including AD. Recently, it was reported that humanized anti-Tau mAb, semorinemab, reduced the rate of cognitive decline as measured by the ADAS Cog 11 (43.6% reduction relative to the placebo arm). Unfortunately, it had no significant effect on the rate of functional decline in mild-to-moderate AD^21,49,50^. Trends toward lower CSF total and phosphorylated Tau levels were seen in the patients treated with semorinemab^51^, suggesting efficient Tau clearance despite lack of efficacy in the advanced stage disease subjects. These results and other preclinical and clinical data on Aβ and Tau immunotherapies^22,26,52,53^ support our long-standing tenet that safe and immunogenic preventive vaccines targeting appropriate B cell epitopes from Aβ and Tau should be initiated in non-impaired subjects. Such early vaccination can inhibit the aggregation of Aβ and Tau and delay the downstream pathological processes (inflammation and neurodegeneration). Unfortunately, passive immunization may be impractical for preventive treatment due to the complexity, cost, and need for monthly intravenous injections of asymptomatic people with a high dose of mAb. By contrast, active vaccines generating sufficient levels of long-lasting immune responses may protect the patient from overt disease and have been used as preventive measures for over a hundred years. Accordingly, we have developed MultiTEP platform technology composed of the string of twelve promiscuous foreign Th epitopes^27,29,36,54–57^ to generate a universal vaccine platform for neurodegenerative disorders. This platform was specifically designed for the elderly with immunosenescence^24,25,58^ and is intended to (i) overcome self-tolerance; (ii) activate not only naïve but also pre-existing memory Th cells^29,53^ specific to pathogens that most adults would have been either exposed to or vaccinated against; (iii) avoid the generation of potentially harmful autoreactive T cells specific to molecules involved in AD pathology; and (iv) be effective in a broad population of humans despite high MHC class II gene polymorphism.

Using the MultiTEP platform technology, we generated recombinant protein- and nucleic acid-based vaccines targeting B cell epitopes of pathological Aβ, Tau, and α-synuclein; and reported on the immunogenicity and efficacy of these vaccines in appropriate disease models^26,27,29,36,54–57,59–61^. In fact, after obtaining the CMC data and completing the IND-enabling safety-toxicology studies with cGMP DNA vaccine targeting pathological Aβ, we received FDA clearance for Phase 1 studies with early AD patients that will be initiated in mid-2022^59^. Finally, we recently demonstrated the immunogenicity and efficacy of the Tau-specific AV-1980R/A in various models of Tauopathy^27,28^ and AD (e.g., bigenic T5x)^26^.

AV-1980R/A vaccine targets B cell epitope of pathological Tau localized in PAD, spanning residues 2–18. It has been shown that the PAD plays a critical role in the aggregation-mediated toxicity of the protein. The increased exposure of the PAD in aggregated Tau leads to dephosphorylation of the kinesins and dropping of the cargo, thus inhibiting the fast anterograde axonal transport. The PAD exposure is also critical in the self-polymerization of hyperphosphorylated Tau, and it was suggested that phosphorylation of Y18, as well as truncation of the N-terminal region of Tau at the late stages of AD, may remove a toxic region and have a protective role. The PAD region of Tau is typically hidden in a paperclip-like conformation of the native protein and becomes exposed during the aggregation^62–67^. Immunohistochemical studies of human postmortem tissues and immunoreactivity with AD brain extracts with PAD-specific commercial mAb (TNT-1) demonstrated that the exposure of the N-terminal region of Tau is an early event in AD that is becoming progressively more prominent in the later stages of AD^62,66–68^. The presence of extracellular N-terminal Tau fragments (eTau) secreted by iPSC cortical neurons from AD patients has been reported ex vivo. Based on these data, the authors suggested that eTau negatively impacts the neurons by inducing their hyperactivity and may elevate Aβ production in the AD brain. Thus, the neutralization of these species can potentially slow the clinical progression of dementia. Based on these and our published data^26–28,30,56^, we hypothesize that anti-PAD antibodies generated by the AV-1980R/A vaccine in cognitively unimpaired people with APOEɛ4 allele and a positive Aβ scan (>20 centiloids) may prevent the accumulation and propagation of pathological Tau protein without binding to the native Tau molecule, and delay the onset of the disease.

The AV-1980R/A was explicitly designed to avoid the activation of autoreactive (Tau-specific) T cells by providing foreign epitopes for T cell activation within the MultiTEP platform. This is critical because the activation of autoreactive T cells could be harmful and cause meningoencephalitis, as in the case of the first anti-Aβ vaccine study (AN-1792)^69^. Here we demonstrate that the AV-1980R/A vaccine activated a broad but individualized repertoire of Th cells specific to the MultiTEP platform without induction of potentially harmful autoreactive Th cells against Tau in the immunized macaques (Figs. 3, 4; Supplementary Table 2). Activated Th cells support the production of high titers of IgG antibodies specific to the _4_PRQEFE_9_ and _7_EFEVMEDH_14_ B cell epitopes of PAD, and these antibodies recognize pathological forms of Tau in the brain sections from AD cases (Figs. 5, 6). The immunogenicity data presented here and our previously published efficacy data^26–28,56^ strongly support testing AV-1980R/A in a preventive Phase 1 clinical trial.

It is unclear which Tau regions or epitopes an immunotherapeutic should target to prevent Tau aggregation and spreading. Currently, various groups and companies are testing in clinical trials antibodies specific to N- and C-terminal, proline-rich, and repeat domains, as well as specific to various phosphorylated forms (Supplementary Table 5). Previously we demonstrated that the anti-PAD antibodies induced by AV-1980R/A vaccination, as well as the mAb armanezumab generated after immunization with this vaccine^27,30^, differ from commercially available anti-PAD mAb, TNT-1. In contrast to TNT-1, immune sera from AV-1980R/A vaccinated animals and mAb armanezumab bind not only Tau monomers and small fragments but also the aggregated forms of Tau in the brain extracts from AD patients. This difference may be due to epitopes recognized by these antibodies. In addition, AV-1980R/A-induced antibodies bind different B cell epitopes of pathological Tau compared with semorinemab, tilavonemab (aka HJ8.5) and gosuranemab. More specifically, immune sera from AV-1980R/A recognize two overlapped epitopes comprising amino acids 3–8 and 7–14, and armanezumab recognizes tau epitope comprising amino acids 3–8, while semorinemab, tilavonemab and gosuranemab recognize residues 6–23, 25–30 and 15–22 respectively^50,70^.

There are also two active vaccination trials with AADVac1 and ACI-35. The former is from AXON Neuroscience and targets residues 294–305 from the second repeat domain;^47,71^ the latter is from AC-Immune and targets Tau phosphorylated at positions 396 and 404 (B cell epitope spanning residues 393–408)^72^. These clinical studies are critical because they might reveal disease-related B cell epitopes that could be used to generate effective preventive vaccines^73,74^. In this regard, if the MultiTEP-based AV-1980R/A vaccine proves safe and immunogenic in humans, this universal platform can be used for targeting any disease-related Tau B cell epitope, including post-translationally modified epitopes^60^. Such a vaccination program could be cost-effective and advantageous, allowing the generation of high titers of high-avidity long-lasting antibodies in cognitively unimpaired people at risk of AD, delaying downstream pathological processes such as inflammation, oxidative stress, and neuronal loss^3,37,40^. Several mechanisms have been implicated in the antibody-mediated reduction of the predominantly intracellular pathological Tau. Some data indicate that antibodies could interact with Tau in the endosomal-lysosomal system and facilitate the clearance of pathological intracellular Tau^75–83^. While the exact mechanism involved in antibody-mediated clearance of intracellular Tau is not known yet, it was shown that abnormally folded and phosphorylated cytoplasmic Tau can be transferred from one neuronal or glial cell to neighboring cells by various mechanisms^84–86^. Such Tau propagation from cells to cells could be inhibited by anti-Tau antibodies preventing the spreading of a pathological molecule within the brain regions and delaying the disease onset and progression^87^. Notably, it was reported that not only pathological^88–91^ but also monomeric native Tau can be released from one neuron and internalized by another^92^. We could only speculate that antibodies generated by immunizations with the MultiTEP platform-based AV-1980R/A vaccine might bind the PAD region of Tau that is usually hidden in a paperclip-like conformation of the native protein but becomes exposed prior to aggregation^93^. We previously reported that the anti-PAD antibodies generated by AV-1980R/A recognized Tau in brain homogenates from AD cases but not in brain homogenates from non-AD cases^27^. More recently, we completed IND enabling studies with AV-1980R/A and demonstrated that this first run cGMP grade vaccine is safe in two different mouse models of AD, in outbred rabbits (paper in prep) and NHP.

Thus, we manufactured the cGMP (first engineering run) Tau vaccine, AV-1980R/A, based on the universal MultiTEP platform and demonstrated that it is highly immunogenic in NHP. These animals have an immune system similar to that of humans. In them, the AV-1980R/A vaccine activates a broad repertoire of MultiTEP-specific Th cells, which then activate B cells producing antibodies specific to the PAD region of Tau. Generated antibodies specific to two overlapped epitopes, Tau_3–9_ and Tau_7-14,_ recognize pathological Tau tangles and Tau-positive neurites in brains from AD cases.

To summarize, the reported here immunogenicity data and the previously published efficacy results for AV-1980R/A^26–28^ support the AV-1980R/A vaccine progression to the first-in-human clinical trials. We hypothesize that vaccination of cognitively unimpaired people at risk of AD with AV-1980R/A can produce antibodies that will bind PAD of extracellular Tau and reduce the propagation of this early pathological molecule in the brain, thus delaying the onset of dementia.

## Methods

### Animals and vaccine administration

#### Monkeys

Six adults (four females, two males), genetically unselected cynomolgus monkeys (Macaca fascicularis) ranging in age from 11 years 4 months to 17 years 8 months from the primate colony at the Alpha Genesis, Inc (Yemassee SC) were injected with 100 µg of AV-1980R formulated in Advax^CpG^ (10 mg advax mixed with 100 µg CpG per dose) adjuvant at weeks 0, 2, 6, 26 and 46. Monkeys were housed according to the accepted standards and observed once daily for abnormal clinical signs or signs of illness or distress, including food intake, activity, appearance, and stool consistency. Body weights were measured before the first vaccine administration and at the time of subsequent administrations and blood draws.

### Design of epitope vaccine

Minigene encoding 3xTau_2-18_-MultiTEP^27^ (Supplementary Fig. 1) was synthesized and cloned into the *E. coli* expression vector pD451-SR:358897 (ATUM, Newark, California). DNA sequencing was performed to confirm that the generated plasmid contained the correct sequences. *E. coli* BLR(DE3) cells were transformed with the pD451-SR-3Tau2-18-MultiTEP (pAV-1980) plasmid, and a research cell bank (RCB) was prepared at the Gates Biomanufacturing Facility (GBF) at the University of Colorado.

### Preparation of Master cell bank (MCB), manufacturing and characterization of AV-1980R

The master cell bank (MCB) was prepared at Charles River Laboratories (Malvern, PA) in compliance with the Good Manufacturing Practice (GMP) requirements of the US Food and Drug Administration (FDA) as found in Title 21 CFR, Parts 210 and 211. AV-1980R engineering run was manufactured at the GBF. The bulk AV-1980R process had been established at a 50 L scale in a cGMP manufacturing facility. The process involved a high-density fed-batch fermentation of an E. Coli BLR(DE3) strain that expresses AV-1980R as inclusion bodies. The cells were lysed and washed several times to isolate the inclusion bodies. The inclusion bodies were then solubilized in a denaturing solution and treated with sodium sulfite and sodium tetrathionate to cleave the disulfide bonds and convert the cysteine thiols to protected S-sulfonates and prevent disulfide bond formation during the following purification steps. The solubilized AV-1980R was precipitated with ammonium sulfate, washed, and then resolubilized in a low ionic strength denaturing solution to prepare for anion exchange bind and elute chromatography. The eluate pool from the anion exchange chromatography was then treated with a reducing agent to remove the sulfonate groups from the cysteines, the pH and conductivity were lowered, and the AV-1980R was further purified using cation exchange chromatography. The product was then allowed to oligomerize during the final formulation using tangential flow filtration buffer exchange to replace the denaturing solution with a phosphate buffer and concentrate the protein to approximately 1.5 mg/mL. Finally, the solution was aseptically filtered through a ≤0.2-micron filter to reduce bioburden. The process generated a nanoparticulate vaccine product of high purity.

### Interferon-γ ELISPOT assay

Analysis of interferon (IFN)-γ-producing PBMCs from immunized Macaca fascicularis were detected by ELISPOT assay as recommended by the manufacturer (Mabtech). PBMC were collected seven days after the third immunization and re-stimulated in vitro with Tau_2-18_; or individual peptides incorporated into MultiTEP: PADRE, P2, P21, P23, P30, P32, HBsAg, HBVnc, MT, P7, P17, and P28; or a cocktail of the MultiTEP peptides; or an irrelevant peptide. Individual peptides were used at a concentration of 20 μg/mL, whereas the cocktail included 4 μg/mL of each peptide. Spots were counted using a microanalyzer (CTL-ImmunoSpot S5; Cellular Technology, Ltd., Shaker Heights, OH, USA). Then we calculated the differences in numbers of spot-forming colonies (SFCs) per 10^6^ PBMCs re-stimulated with MultiTEP peptides or Tau_2-18_ and subtracted the number of SFC per 10^6^ PBMCs detected after restimulation with the irrelevant peptide (baseline).

### Detection of Tau-specific antibodies and fine mapping of B-cell responses

The concentrations and endpoint titers of anti-Tau antibodies in macaque sera were determined by enzyme-linked immunoassay (ELISA) as described previously^29,36,54^. Briefly, plates were coated with Tau_2-18_ peptide (GenScript), then washed and blocked. Serial dilutions of immune sera were added to the wells. After incubation and washing, an appropriate HRP-conjugated IgG (goat anti-monkey IgG, cat # PIA-84631, Invitrogen, MA, USA) was used as a secondary antibody at 1:2000 dilution. Plates were incubated and washed, and the reaction was developed by adding 3,3ʹ,5,5ʹ-tetramethylbenzidine/H_2_O_2_ (TMB) (Pierce, IL, USA) substrate solution and stopped with 2N H_2_SO_4_. The optical density was read at 450 nm (FilterMax F5). Endpoint titers of antibodies in monkey sera were calculated as the reciprocals of the highest sera dilutions that gave an optical density reading trice above the cutoff. The cutoff was determined as the titer of pre-immune sera at the same dilution. Concentrations were calculated using a calibration curve generated with mAb armanezumab (Institute for Molecular Medicine, Huntington Beach, CA, USA).

The isotypes of monkey anti-Tau antibodies were evaluated in serum diluted at 1:2000, using horseradish peroxidase (HRP)-conjugated anti-monkey IgG (Cat #43R-IG020HRP, Fitzgerald Industries, Inc., Acton, MA, USA) and anti-monkey IgM (Cat # 70031, Alpha Diagnostic Intel, Inc., San Antonio, TX, USA) secondary antibodies at dilutions of 1:50,000 and 1:2000, respectively.

Epitope mapping of anti-Tau antibodies induced by the vaccination in monkeys was performed by alanine scanning using competitive ELISA. Briefly, 17 peptides spanning Tau_2–18_ sequence with singular alanine substitutions in each position were synthesized. Ninety-six-well ELISA plates (Immulux HB; Dynex Technologies, Inc., VA) were coated with 1 μg/well (in 100 μl; Carbonate-Bicarbonate buffer, pH 9.6, o/n at 4 °C) Tau_2-18_ peptide (GenScript, NJ). The next day, coated plates were blocked with blocking buffer (3% dry, non-fat milk in TBST, 300 μl/well). Serial dilutions of the reference wild-type (Tau_2-18_) or alanine-substituted peptides (corresponding to 0 μM, 0.02 μM, 0.1 μM, 0.5 μM, 2.5 μM, 5 μM, and 12.5 μM final concentrations) were incubated with 0.08 mg/ml antibody purified from the pooled monkey sera (corresponding to the linear region of the curve for binding to Tau_2-18_ peptide) for 1.5 h at 37 °C. After incubation, 100 μl of antibody/peptide mixture was added to the wells. HRP-conjugated goat anti-monkey IgG (1:2000; Invitrogen) were used as secondary antibodies. The reaction was developed using TMB substrate solution and stopped with 2 M H_2_SO_4_. The optical density (OD) was read at 450 nm (Biotek, Synergy HT, VT). The percent of binding of antibodies blocked with wild-type or alanine-substituted peptides to Tau_2-18_ was calculated relative to the binding of antibodies without competing peptides to Tau_2–18_ as 100%. The half-maximal inhibitory concentration (IC50) for each peptide was calculated.

### Detection of anti-Tau antibody binding avidity by enzyme-linked immunosorbent assay (ELISA)

Sodium thiocyanate displacement ELISAs were performed using the method described by Richmond et al.^94^. Antibodies were purified from the pooled sera of mice/rabbits/macaques, and the concentrations were equalized to 30 ng/ml. Bound antibodies were detected as described above, with an exception: after incubation with primary antibodies, plates were washed three times with Tris-buffered saline containing 0.5% Tween-20, then Tris-buffered saline buffer containing 0, 0.5, 1, 1.5, 2, 2.5, 3.5, 4 M NaCSN was added and incubated for 15 min, and washed six more times with Tris-buffered saline containing 0.5% Tween-20. The results were expressed as a percentage of binding in the absence of NaSCN.

### Detection of neurofibrillary tangles in human brain tissues

Macaque’s pre-bleed and immune sera (1:1000) were screened for the ability to bind to human neurofibrillary tangles (NFTs) using 50-mm brain sections of formalin-fixed cortical tissue from an AD subject (Brain Bank and Tissue Repository, MIND, University of California, Irvine) using immunohistochemistry as described previously^55^. KPL HRP-anti-monkey IgG secondary antibodies (Cat # 5220-0333 (074-11-021), SeraCare, Milford, MA, USA) and a digital camera (Olympus, Center Valley, PA) were used to visualize and capture images of the NFTs at 40X magnification.

### Reporting summary

Further information on research design is available in the Nature Research Reporting Summary linked to this article.

## Supplementary information

Supplementary Materials

REPORTING SUMMARY

## Data Availability

All data generated or analyzed during this study are included in this article, and materials are available from the corresponding author upon reasonable request.
