# Supplementary material for: Immunogenicity of MultiTEP platform technology-based Tau vaccine in non-human primates
Source: NPJ Vaccines. 2022 Oct 12;7:117. doi: 10.1038/s41541-022-00544-3 (PMC9556597; doi:10.1038/s41541-022-00544-3)
Supplement: Supplementary file 1 — Supplementary Materials [file 41541_2022_544_MOESM1_ESM.pdf]

B cell epitopes **String of T helper cell epitopes (MultiTEP platform)**

$Tau_{2\text{-}18}$-$Tau_{2\text{-}18}$-$Tau_{2\text{-}18}$-PADRE-P23-P32-P21-P30-P2-HBVnc-HbsAg-MT-P7-P17-P28

- GS linker

Supplementary Figure 1. Schematic representation of AV-1980 construct encoding 3 copies of tau2-18 fused to MultiTEP, one universal synthetic T epitope, PADRE and eleven foreign promiscuous T epitopes from infectious agents. Epitopes in the construct are linked with glycine-serine (GS) linker.

Supplementary Table 1. Parameters showing the stability of AV-1980R at various temperatures and time points.

Time Point	DLS		SDS-PAGE, % of Main Band		Concentration via A280	RP-HPLC					
	Diameter (nm)	% Mass	Reduced	Non-Reduced	Averaged Value (mg/mL)	Retention Time min.	Main Peak (%)	Early Peaks (%)	Pre-Main Peaks (%)	Post Peaks (%)	Pre-Main Shoulder (%)
Release	18.17	99.2	84.5	63.1	1.53	20.4	69.4	2.3	12.2	16.1	-
T=24hr 25°C	21.04	99.6	84.5	55.2	1.52	20.53	50.18	2.26	30.47	17.08	-
T=72hr 25°C	21.04	99.3	80.3	49.0	1.55	20.55	51.78	2.29	26.74	19.19	-
T=0.5 month 5°C	18.17	98.8	88.3	51.8	1.44	20.53	68.35	2.5	11.45	17.7	-
T=1 month 5°C	21.04	99.6	85.9	45.6	1.61	20.52	55	2.48	11.99	16.36	14.18
T=1 month -20°C	24.36	99.3	85.9	68.6	1.55	20.48	57.45	2.54	11.85	14.89	13.27
T=3 month -20°C	22.15	100	83.8	69.2	1.59	20.48	56.58	2.36	11.73	14.50	14.83
T=3 month -80°C	19.13	99.3	84.5	63.1	1.51	20.52	49.68	2.36	31.54	16.42	-
T=6 month -80°C	21.04	99.7	83.0	59.7	1.55	20.47	58.43	2.37	11.86	14.40	12.93
T=6 month -20°C	24.36	99.7	94.1	70.2	1.50	20.37	59.96	2.32	11.70	16.40	9.62
T=9 month -80°C	21.04	99.9	94.5	82.6	1.52	20.35	61.13	2.37	12.07	15.25	9.18
T=9 month -20°C	25.6	99.8	85.8	66.6	1.44	20.35	55.42	2.44	12.17	12.75	17.21
T=12 month -80°C	19.13	99.2	82.6	71.1	1.47	20.26	70.12	2.32	11.63	15.93	0.00
T=12 month -20°C	25.6	99.9	86.8	46.1	1.38	20.33	58.36	2.48	12.25	12.85	14.05
T=15 month -80°C	21.0	99.9	88.0	55.1	1.4	20.30	44.20	2.38	12.13	15.61	25.68
T=18 month -80°C	21.0	99.8	88.2	67.84	1.43	20.4	50.4	2.3	11.49	13.9	21.9

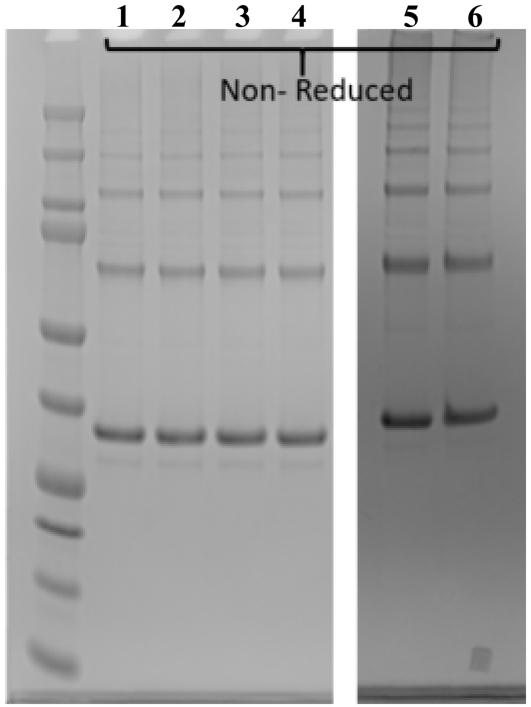

1. **24hrs, RT, AV-1980R+PBS**
2. **24hrs, RT, AV-1980R+Advax**
3. **24hrs, RT, AV-1980R+CpG**
4. **24hrs, RT, AV-1980R+Advax^{CpG}**
5. **72hrs, RT, AV-1980R+PBS**
6. **72hrs, RT, AV-1980R+ Advax^{CpG}**

Supplementary Figure 2. Stability of AV-1980R formulated in Advax^{CpG} adjuvant. Sample was thawed after 1 month storing at -80°C, incubated with PBS, Advax, CpG or Advax^{CpG} and analyzed in SDS-PAGE in non-reduced conditions.

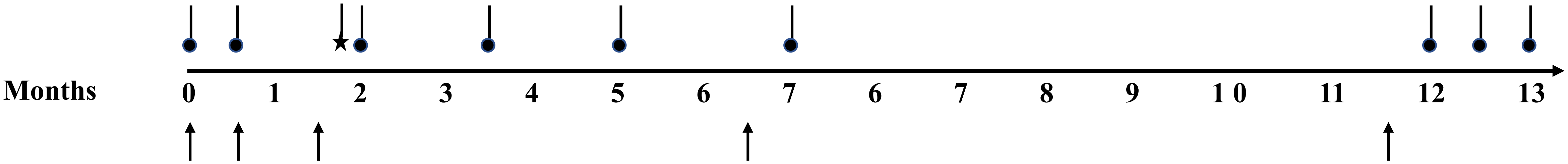

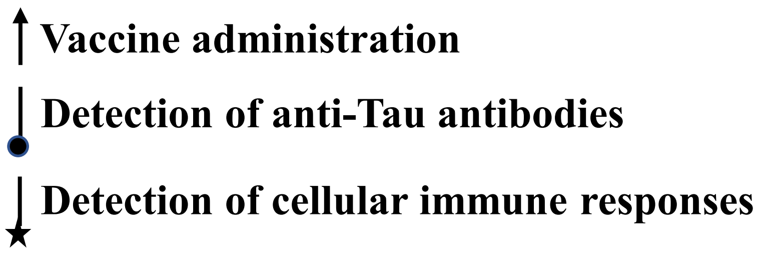

NHP ID number	Sex	Age (year)	Immunogen	Dose
5C4-34	F	12.6	AV-1980R/A	100 µg
DR5K	F	11.4	AV-1980R/A	100 µg
CH2C	F	17.8	AV-1980R/A	100 µg
CT5G	F	17.0	AV-1980R/A	100 µg
CM9P	M	16.9	AV-1980R/A	100 µg
H2D	M	17.8	AV-1980R/A	100 µg

Supplementary Figure 3. Design of experimental protocol in NHPs vaccinated with AV-1980R/A.

Supplementary Table 2. Mapping of Th epitopes in NHPs immunized with AV-1980R/A

Monkey No/vaccine	Th epitope											
	PADRE	P23	P32	P21	P30	P2	HBVnc	HBsAg	Flu MT	P7	P17	P28
CM9P/AV-1980R/A	-	+/-	-	-	-	+/-	-	-	-	-	+	+/-
CH2D/AV-1980R/A	+/-	-	-	-	-	-	-	-	-	-	+	+/-
CT5G/AV-1980R/A	+	+	+/-	-	+	+	+/-	-	+	+	+	+
CH2C/AV-1980R/A	+	+	-	+/-	-	+	+	+	+/-	+/-	+/-	-
DR5K/AV-1980R/A	+/-	+	+/-	+/-	+	+	+	+	+	+/-	+	+
5C4-34/AV-1980R/A	+	+	+	+	+	+	+	+	+	+	+	+
% of Responders	83	83	50	50	50	83	67	50	67	67	100	83

The number of IFNγ producing T cells/10^6 splenocytes is < 40 (-); 40-100 (+/-); ≥100 (+)

Supplementary Table 3. Select list of tau-targeted mAb and vaccines in clinical trials

mAb/vaccine	Company	Region	Epitope	Status	Participants	Reference
Semorinemab	Roche/AC	N-terminal Projection domain	aa 10-24	Phase II	AD	Ayalon G.et al., Sci Transl Med., 2021 https://www.alzforum.org/therapeutics/semorinemab
ABBV-8E12 Tilavonemab	AbbVie/C2N Diagnostics	N-terminal Projection domain	aa 22-34	Phase II	PSP, AD	Höglinger G.U. et al., Lancet Neurol, 2021 https://www.alzforum.org/therapeutics/tilavonemab
Gosuranemab	Biogen/Bristol Myers Squibb	N-terminal Projection domain	aa 15-22	Phase II	PSP, AD	Dam T. et al., Nature Med, 2021; Boxer AL, Lancet Neurol.2019
BIIB076	Biogen	N-terminal Projection domain	aa 125-131	Phase I	Healthy, AD	Nobuhara C.K. et al., *Am J Pathol*. 2017 https://www.alzforum.org/therapeutics/biib076
Beprenemab	Hoffmann-La Roche, UCB S.A.	Proline rich domain/RD1	aa235-250	Phase I	Healthy volunteers, PSP	https://www.alzforum.org/therapeutics/bepranemab
E2814	Eisai	RD2	aa 299-303	Phase I	Healthy volunteers	Roberts M. et al., Acta Neuropathol Commun. 2020
JNJ-63733657	Johnson & Johnson	Proline rich domain	p212 p217	Phase II	AD	Galpern W.R. et al., AAIC 2019 https://www.alzforum.org/therapeutics/jnj-63733657
Lu AF87908	H. Lundbeck	C-terminal domain	p396/p404	Phase 1	Healthy volunteers, AD	CTAD 2020 https://www.alzforum.org/therapeutics/lu-af87908
Zagotenemab	Eli Lilly	Conformational	Binding to aa 7-9 and 312-322	Phase II	Healthy volunteers, AD	https://www.alzforum.org/therapeutics/zagotenemab
ACI-35	Johnson & Johnson/ AC Immune	C-terminal domain	p396 /p404	Phase I/II	AD	https://www.alzforum.org/therapeutics/aci-35
AADVac1	AXON Neuroscience	RD2	aa 294-305	Phase 2	AD	Novak P. et al., Nature Aging, 2021 https://www.alzforum.org/therapeutics/aadvac1
